# *Lactobacillus brevis* Strains from Fermented *Aloe vera* Survive Gastroduodenal Environment and Suppress Common Food Borne Enteropathogens

**DOI:** 10.1371/journal.pone.0090866

**Published:** 2014-03-05

**Authors:** Young-Wook Kim, Young-Ju Jeong, Ah-Young Kim, Hyun-Hee Son, Jong-Am Lee, Cheong-Hwan Jung, Chae-Hyun Kim, Jaeman Kim

**Affiliations:** 1 KBNP Technology Institute, KBNP Inc., Yesan, Korea; 2 School of Biological Sciences and Technology, Chonnam National University, Gwangju, Korea; 3 Department of Biology, Mokpo National University, Muan, Korea; University of Malaya, Malaysia

## Abstract

Five novel *Lactobacillus brevis* strains were isolated from naturally fermented *Aloe vera* leaf flesh. Each strain was identified by Random Amplified Polymorphic DNA (RAPD) analysis and 16S rRNA sequence comparison. These strains were highly tolerant to acid, surviving in pH2.5 for up to 4 hours, and resistant to 5% bile salts at 37°C for 18 hours. Due to its tolerance to acid and bile salts, one strain passed through the gastric barrier and colonised the intestine after oral administration. All five strains inhibited the growth of many harmful enteropathogens without restraining most of normal commensals in the gut and hence named POAL (Probiotics Originating from Aloe Leaf) strains. Additionally, each strain exhibited discriminative resistance to a wide range of antibiotics. The *L. brevis* POAL strains, moreover, expressed high levels of the glutamate decarboxylase (GAD) gene which produces a beneficial neurotransmitter, γ-aminobutyric acid (GABA). These characteristics in all suggest that the novel *L. brevis* strains should be considered as potential food additives and resources for pharmaceutical research.

## Introduction

The *Aloe vera* L. plant has been used widely in herbal medicines for millennia [1] and it is a significant resource for cosmetic and pharmacological industries. It is one of a few edible species among approximately 500 species of *A. vera*. Its fleshy leaves have phenolic compounds such as aloe emodin, aloin, and aloesin, organic acids such as saponins and terpenoids [8]. With these ingredients, *Aloe vera* extracts have antibacterial and antifungal activities, which may be able to treat minor skin infections, such as boils and benign skin cysts [33 34]. *Aloe vera*, inner-leaf gel inhibits growth of *Streptococcus* and *Shigella* species *in vitro* [15]. In spite of these antimicrobial activities, the leaf gel is prone to oxidation at various temperatures, which leads to fermentation. This phenomenon is due to degradation of phenolic compounds in the aloe, which might be related a plant defense mechanism [11]. Oxidation of these compounds ablates the antimicrobial activity and results in bacterial growth. Preliminary investigation of aloe flesh fermentation revealed the presence of lactic acid bacteria (LAB). Therefore, we hypothesised that LAB produce compounds with antimicrobial activities in the aloe flesh and that these bacteria proliferate after aloe’s antimicrobial compounds deteriorate.

Plant-derived LABs are generally found in fermenting fruits, vegetables or traditional foods and pickles in Asia. Plant-derived LABs were first investigated in Japan [40]. These studies focused on the isolation of acid and bile tolerant LAB because plant-derived LAB are much more resistant to artificial gastric juices and bile than animal-derived LAB [7], [36]. In Korea, various acid-tolerant LAB strains have been isolated from kimchi, a traditional Korean food [4], [19], [21], fermented soybean paste [20] and pickles [17].

LABs are widely used in various industries as food and cosmetic additives [24], [31]. These bacteria are “*probiotics*”, which are viable bacilli of a single strain or mixture of strains that are beneficial to human and animals; they improve the composition of intestinal microflora when consumed as dried cells or in fermented products [2], [3], [6], [12]. “*Probiotic*” bacteria must not only survive in the distal ileum and colon but also adhere and colonise there to effectively confer benefits on the host [5], [9], [32]. However, the low-pH and antimicrobial environment of the stomach forms a natural barrier to bacterial entry into the intestinal tract. Therefore, acid tolerance is one of the main criteria for selecting of “probiotics” [14].

The aims of this study were to isolate LAB from naturally fermented *A. vera* L. leaf flesh and to prove their probiotic properties, i.e., antimicrobial activity and tolerance to acid and bile salt. These putative probiotic bacteria and their metabolites may be useful resources for the development of antibiotics and antimicrobial substances. Moreover, probiotic bacteria may enhance the therapeutic value of *A. vera* L products.

## Materials and Methods

### Ethics Statement

No specific ethics permits were required for the described studies. The collection of *A. vera* L. plants were purchased from the private farm owners of Jayeon aloe (Jindo Island, Jeonnam, Korea), under Licence Number 415/06/88966 of Korea Food and Drug Administration (KFDA). Collections were not performed in national parks or other protected area of land, and did not involve endangered or protected species. All animal experiments complied with the current laws of Korea. Animal care and treatment were conducted in accordance with guidelines established by the Institute Institutional Animal Care and Use Committee of Chonnam National University. The protocol was approved by the committee on the Ethics of Animal Experiments of the Chonnam National University.

### Isolation of Acid-tolerant LAB

Leaves were collected from *A. vera* L. plants that had been grown for more than five years and allowed to ferment naturally for 20–30 days at RT. Fermented substances(pH 3.5–3.8) were centrifuged, and the supernatant was transferred to a sterile tube. MRS broth adjusted to pH 2.5 was inoculated with the fermentation supernatants (1% v/v final concentration). After 5 h incubation at 37°C, acid-resistant colonies were selected by the agar dilution method. Briefly, ten-fold serial dilutions of the MRS broth culture were spread onto MRS agar (Difco, USA), and plates were incubated at 37°C for 24 h in aerobic conditions. Isolated colonies were randomly picked for further selection.

A total of 113 colonies were subjected to a bromocresol purple (BCP) test. After 24 h incubation at 37°C, yellow colonies were selected as the positive clones of LAB [26]. From a total of 113 colonies, 65 colonies were subjected to a haemolysis test modified from [38]. This procedure ruled out 35 haemolytic colonies, and the other 30 colonies were investigated further to identify individual strains. The isolated colonies were stored at −80°C in MRS broth containing 10% glycerol.

### Identification of Individual Strains

Each colony was streaked on an MRS agar plate and incubated at 37°C for 24 h in aerobic conditions. A single colony from the plate was transferred to MRS broth and incubated for 18 h at 37°C. To prepare genomic DNA and rRNA, bacteria were lysed with lysozyme and mutanolysin as described previously [39], and genomic DNA was extracted using an Accuprep Genomic DNA Extraction Kit (Bioneer, Korea).

RAPD analysis was carried out with 20 OPA and 20 OPC random primer sets (Operon, USA). PCR was performed with HiPi 5×PCR premix according to the manufacturer’s instructions(Elpisbio, Korea). DNA templates (10 ng) and primers (5 pmol) were added to the PCR premix and DNase-free distilled water was added to a final volume of 20 µl. Amplification was performed in a thermal cycler (Kyratech, Australia) programmed as follows: 94°C for 5 min; 40 cycles of 94°C for 1 min, 38°C for 1 min, and 72°C for 1.5 min; and 72°C for 5 min. PCR products were analysed on a 2% agarose gel. All procedures were repeated at least three times to ensure reproducibility. RAPD data were analysed with MVSP-32 software (Kovach Computing Services, UK) to calculate the genetic similarity between isolates and to draw a phylogenetic dendrogram by the UPGMA method.

The 16S rRNA gene was amplified using the following universal primers: (forward), 5′-AGAGTTTGATCCTGGCTCAG-3′and (reverse) 5′-GGTTACCTTTGTTA CGACTT-3′ (Bioneer, Korea). The thermal cycling parameters are as follows: denaturation at 94°C for 5 min, 30 cycles of 94°C for 1 min, 55°C for 1 min, and 72°C for 40 s and a final extension at 72°C for 5 min. Amplified products were separated on an agarose gel and purified for sequencing with a Gel Extraction Kit (Intron, Korea). The 16S rRNA gene products were subcloned into the pGEM-T easy vector (Promega, USA) and sequenced by Bioneer company (Korea). Sequence homologies were determined by BLASTn searches of the NCBI database (http://www.ncbi.nlm.nih.gov/BLAST) and by Molecular Evolutionary Genetics Analysis with maximum likelihood and neighbor-joining methods (Mega 4.0).

### Characterisation of LAB Strains

The following reference strains were obtained from the Korean Collection for Type Cultures (KCTC, Daejeon, Korea), the Korean Culture Center of Microorganisms(KCCM, Seoul, Korea) and American Type Culture Collection(ATCC, Rockville, MD, USA): *B. cereus* KCTC1012^T^, *C. perfringens* KCTC3269^T^, *C. jejuni* ATCC33560^T^, *E. coli* ATCC33694^T^, ATCC8739^T^ and ATCC43888^T^, *E. aerogenes* KCTC2190^T^, *E. faecalis* KCTC2011^T^, *K. pneumonia* KCTC2208^T^, *L. monocytogenes* KCTC3567^T^, *P. acidilactici* KCTC1626^T^, *P. aeroginosa* KCTC1750^T^, *S. aureus* KCTC1621^T^, *S. typhi* KCTC2424^T^, *S. enteroritidis* KCCM12400^T^, and *L. brevis* KCTC 3498^T^, KCTC 3102^T^.

Carbohydrate utilisation was investigated with an API kit (API 50 CHL) from Biomerieux (Marcy-l’Etoile, France) according to the manufacturer’s instructions, and the results were analysed according to Biomerieux’s guide.

For acid tolerance tests, the selected strains were subjected to a more accurate screening for acid tolerance in growth media, adjusted to pH 2.5, or 3.0, with 0.5 M HCl and SGF buffer [simulated gastric acid fluid,; 2 g/L NaCl, 3.2 g/L pepsin (Sigma, USA)]. Bacteria were grown on each type of media for the indicated time at 37°C. Afterwards, cell viability was determined by the agar dilution method on BCP agar plate. The original cell density (OCD) was calculated using the formula; OCD = [colonies on plate]/[volume of sample plated × dilution factor]. The survival rate was calculated as the percentage of OCD on MRS. Each experiment was performed in triplicates.

POAL strain bile salt tolerance was examined in 10 ml of MRS broth supplemented with 0.3, 0.5, 1.0, 2.0, and 5.0% (w/v) oxgall bile salt (Difco, USA) or a negative control (0% bile salt); cultures were incubated for 18 h at 37°C. Bacterial cultures were serially diluted in PBS (pH 7.4) and plated on BCP agar. After incubation for 24 h at 37°C, colonies were counted. The survival rate at each concentration of bile salt was calculated as the ratio of colonies to those on the negative control plate. The experiment was performed in triplicates.

Antibiotic susceptibility was tested by the agar dilution method published by the National Committee for Clinical Laboratory Standards [25]. To determine the minimum inhibitory concentration (MIC) of antibiotics, concentrations recommended by the Scientific Committee on Animal Nutrition (SCAN) were used. The antibiotic concentrations (per ml of MRS agar) were as follows: neomycin (30 µg), streptomycin (10 µg), Amoxicillin/Clavulanic Acid (20/10 µg), Ampicillin (10 µg), Cefotaxime (30 µg), Chloramphenicol (30 µg), Ciprofloxacin (5 µg), Colistin (10 µg), Doxycycline (30 µg), Erythromycin (15 µg), Gentamicin (10 µg), Tetracycline (30 µg), and Trimethoprim/Sulfamethoxazole (1.25/23.75 µg). Antibiotic discs were obtained from Difco (USA). The POAL strains were subcultured twice before susceptibility tests. MRS agar plates were inoculated with approximately 10^9^–10^10^ CFU/ml (final concentration; 100 µl final volume per well) and incubated for 24 h at 37°C before MICs were visually determined. Inhibition zones were measured and strains were categorised as resistant (R), intermediate (I), or susceptible (S).

#### Antibacterial activities

Antibacterial activities of the POAL strains against several Gram-positive and Gram-negative strains were tested using the penicylinder method, originally described by [13]. Overnight cultures of the indicator strains were diluted to a concentration of 10^5^–10^6^ CFU/ml, transferred into nutrient agar and brain heart infusion agar (Difco, USA) at 37°C, and poured into plates. Two stainless steel penicylinders (Fisher Scientific, USA) were evenly spaced onto each plate. The penicylinder slots were filled with 150 µl POAL strains, and the plates were incubated at 37°C for 24 h in aerobic conditions. The antibacterial activity of each strain was determined by the size of the inhibition (clear) zone.

#### Intestinal survival of POAL strains

To evaluate the survival of POAL strains in vivo, bacteria were cultured in MRS broth for 24 h at 37°C and pelleted at 3,000×g for 10 min. The bacterial pellets were resuspended in PBS buffer (pH 7.4) and administered orally to each group of female BALB/c mice (n = 3/group) for 1 week. Female BALB/c mice (5–6 weeks; 24±1.5 g) were purchased from the Yang-Sung Experimental Animal Center (Korea) and housed in standard environmental conditions (12 h light–dark cycle, 50–70% humidity and 20–25°C). Food and water were provided *ad libitum*. The mice were sacrificed by the cervical dislocation. Abdominal cavities of mice were cut open by a midline abdominal incision to collect large intestines. Large intestines were obtained from test mice and flushed with PBS buffer (pH 7.4). The lavage fluids were diluted 10-fold, incubated in SGF buffer (pH 2.5) at 37°C for 4 h, plated on MRS agar (Difco, USA) containing 30 µg of tetracycline at 37°C for 24 h. The strain identities of selected colonies were determined by RAPD-PCR. Each experiment was performed in triplicate.

#### Quantification of GAD expression

Total RNA was isolated from POAL strains and two reference strains (KCTC3498^T^, KCTC3102^T^) with a Qiagen RNeasy kit (Qiagen, USA). The isolated RNA was treated with RNase-free DNase (Roche, Switzerland) to remove contaminant DNA. Reverse transcription was performed with 1 µg total RNA and 20 µl reaction mix from an iNtRON Power cDNA synthesis Kit (Intron, Korea) and AMV reverse transcriptase (Intron, Korea), according to the manufacturer’s directions. Real-time PCR amplification was performed with gene-specific primers and SYBR Green Master Mix (Qiagen, USA) using a Rotor-Gene 6000 real-time amplification operator (Corbett Research, Australia). The gene specific primers were *GAD128_F* (5′-GATGAAGTTTGCTTGGCGTAAG-3′) and *GAD128_R* (5′-CGATGTCCC AATAGACACAGAA-3′). *gapB* was used as the reference gene and was amplified with *gapB_F* (5′-ACGGAATTAGTTGCAATCTTAGAC-3′) and *gapB*_R (5′-GAAAGTAGTACC GATAACATCAGA-3′) primers. The PCR conditions were as follows: 1 cycle of 95°C for 15 min and 45 cycles of 95°°C for 25 s, 56°°C for 25 s, and 72°°C for 30 s. Samples were run three times in triplicate, and the relative amount of target RNA for each sample was calculated by statistical analysis [30].

### Statistical Analyses

The results are mean values and standard deviations of 3 measurements from 2 independent assays. Statistical analysis was performed using Sigma Plot 2000 software (SPSS, USA). The results were compared by an Analysis of Variance (ANOVA) general linear model followed by Tukey’s post-hoc test. Statistical significance was determined as p<0.05.

## Results

### Isolation of LAB

From the fermented *Aloe vera* flesh, 113 colonies that survived 4 h incubation at pH 2.5 were isolated (Table 1). The LABs were identified on an MRS agar plate containing bromocresol purple, and yellow colonies were scored as positive. Of the 113 colonies, 65 lactic acid bacteria colonies were screened for haemolytic activity. Thirty colonies were non-haemolytic.

**Table 1:** Screening of LAB from fermented aloe flesh.

Selection step	Number of colonies selected
1. Acid tolerance (pH 2.5 for 4 h at 37°C)	113
2. Bromocresol Purple (Lactic acid positive)	65
3. Haemolysis test (non-haemolytic)	30
4. RAPD Analysis	5 strains*

*5 strains were identified by RAPD analysis.

#### Identification of Five LAB StrainsRAPD analysis

**Figure 1:**
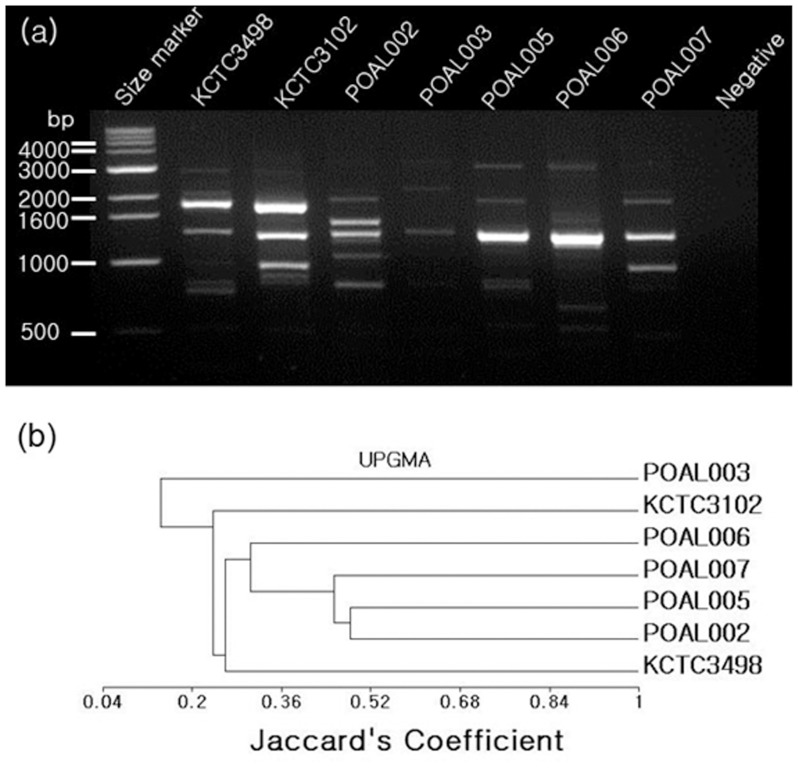
Phylogenetic analysis of isolated *L. brevis* strains by RAPD. (a) A representative RAPD analysis with primer OPA-08 (b) UPGMA phylogenetic tree of isolated strains.

RAPD analysis of the 30 non-haemolytic colonies revealed five lactic acid bacterial strains (Figure 1a). Analysis of patterns produced with 40 RAPD primers resulted in a UPGMA phylogenetic tree (Figure 1b), which showed the relative genetic similarity between the 5 strains. All 5 strains were identified as *Lactobacillus brevis* in the API 50 CHL test (Biomereiux, France), and strains POAL002, 007, 005, and 006 were similar to the reference *L. brevis* strain, KCTC3498^T^. POAL006 was most closely related to KCTC3498^T^. POAL003 strain was least genetically similar to KCTC3498^T^ but was related to another *L. brevis* reference strain, KCTC3102^T^.

#### 16S rRNA sequence comparisons

**Figure 2:**
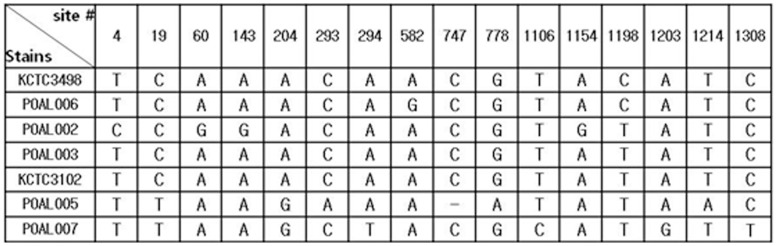
Base changes in the 16S rRNA sequences of *L. brevis* strains.

The genetic identities and phylogenetic relationships of the five strains were confirmed by 16S rRNA gene sequence analysis (Figure 2). The 16S rRNA sequences from all 5 strains were 97–99% similar to *L. brevis* and were most similar to the *L. brevis* ATCC 14869^T^ strain. Thus, these strains were named *L. brevis* POAL 002, 003, 005, 006, and 007 deposited in Genebank with the accession numbers JX185493, JX185494, JX185495, JX185496, and JX185497, respectively). 16S rRNA sequence analysis confirmed the phylogenetic relationships determined by RAPD analysis (Figure 1b).

### Characterisation of the Strains

#### Carbohydrate utilization

Carbohydrate utilisation by the POAL strains was consistent with that of *L. brevis* species (Table 2). The metabolic characteristics of POAL005 were most similar (97.4%) to *L. brevis*, followed by POAL006 (89.1%), POAL007 (88.1%), POAL002 and POAL003 (88%) strains. The reference strains KCTC 3498^T^ and KCTC 3102^T^ were also analysed to validate the accuracy of the assay.

**Table 2:** Biochemical and physiological characteristics of lactic acid bacteria*.

	POAL 002	POAL 003	POAL 005	POAL 006	POAL 007	KCTC 3498	KCTC 3102
Glycerol	−	−	−	−	−	−	−
Erythritol	−	−	−	−	−	−	−
D-Arabinose	−	−	−	−	−	−	−
L-Arabinose	+	+	+	+	+	+	?
Ribose	+	+	+	+	+	+	?
D-Xylose	+	+	+	+	+	+	+
L-Xylose	−	−	−	−	−	−	−
Adonitol	−	−	−	−	−	−	−
β-Methyl-xyloside	−	−	−	−	−	−	−
Galactose	−	−	−	−	−	−	−
D-Glucose	?	?	?	?	?	?	−
D-Fructose	?	?	?	?	?	+	?
D-Mannose	−	−	−	−	−	−	−
L-Sorbose	−	−	−	−	−	−	−
Rhamnose	−	−	−	−	−	−	−
Dulicitol	−	−	−	−	−	−	−
Inositol	−	−	−	−	−	−	−
Mannitol	−	−	−	−	−	−	−
Sorbitol	−	−	−	−	−	−	−
α Methyl-D-mannoside	−	−	−	−	−	−	−
α Methyl-D-glucoside	−	−	−	−	−	−	−
N acetyl glucosamine	?	?	?	−	?	?	?
Amygdaline	−	−	−	−	−	−	−
Arbutine	−	−	−	−	−	−	−
Esculin	−	−	−	−	−	−	−
Salicine	−	−	−	−	−	−	−
Cellobiose	−	−	−	−	−	−	−
Maltose	+	+	?	?	?	+	+
Lactose	−	−	−	−	−	−	−
Melibiose	−	−	−	−	−	−	−
Saccharose	−	−	−	−	−	−	−
Trehalose	−	−	−	−	−	−	−
Inuline	−	−	−	−	−	−	−
Melezitose	−	−	−	−	−	−−	−
D-Raffinose	−	−	−	−	−	−	−
Amidon	−	−	−	−	−	−	−
Glycogen	−	−	−	−	−	−	−
Xylitol	−	−	−	−	−	−	−
β Gentiobiose	−	−	−	−	−	−	−
D-Turanose	−	−	−	−	−	−	−
D-Lyxose	−	−	−	−	−	−	−
D-Tagatose	−	−	−	−	−	−	−
D-Fucose	−	−	−	−	−	−	−
L-Fucose	−	−	−	−	−	−	−
D-Arabitol	−	−	−	−	−	−	−
L-Arabitol	−	−	−	−	−	−	−
Gluconate	?	?	−	?	?	?	−
2 ceto-gluconate	−	−	−	−	−	−	−
5 ceto-gluconate	?	?	?	?	?	?	−

*(−) = negative result, (+) = positive result, (?) = doubtful result, as described in Materials and Methods.

#### Acid tolerance

**Figure 3:**
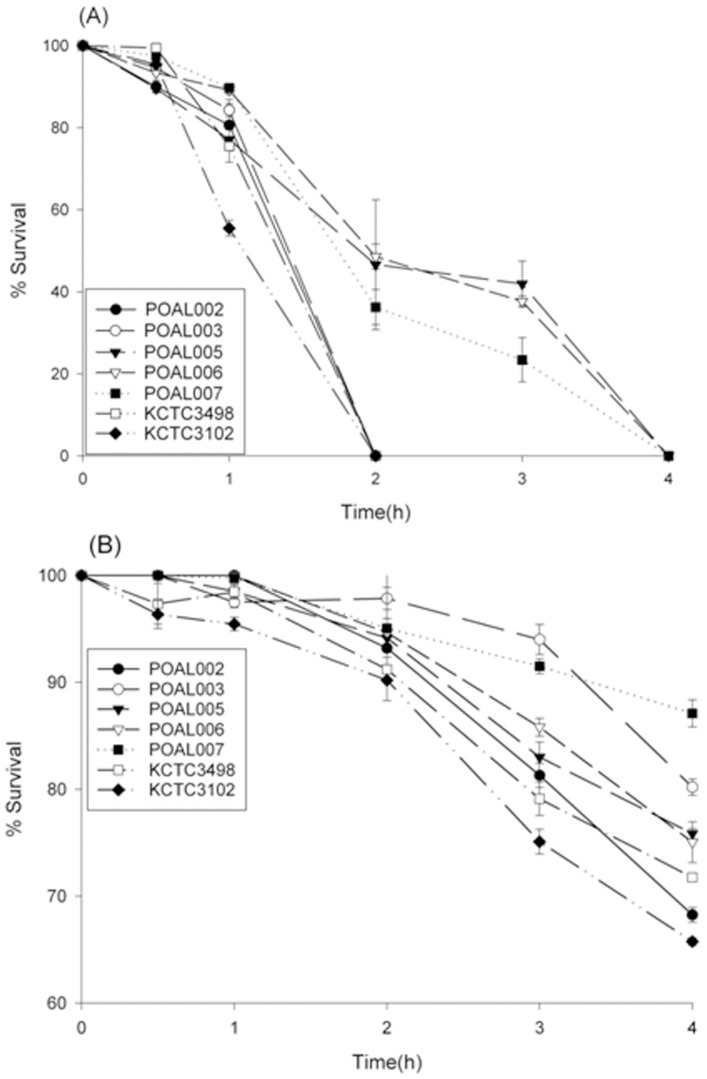
Survival of *L. brevis* POAL strains at pH 2.5 (A) and pH 3.0 (B), respectively.

All the POAL strains were strongly tolerant to acidic conditions (Figure 3). The POAL strains could withstand exposure to pH 2.5 and pH 3.0 conditions better than KCTC 3498^T^, and KCTC 3102^T^. All the POAL strains survived at pH 3.0 for 4 hours. The POAL005, 006, 007 strains were especially acid-tolerant. While the other strains did not survive longer than 2 hours, these 3 strains survived for 4 hours at pH 2.5. The POAL003 strain was superior in particular at pH 3.0 but not at pH 2.5.

#### Bile salt tolerance

**Figure 4:**
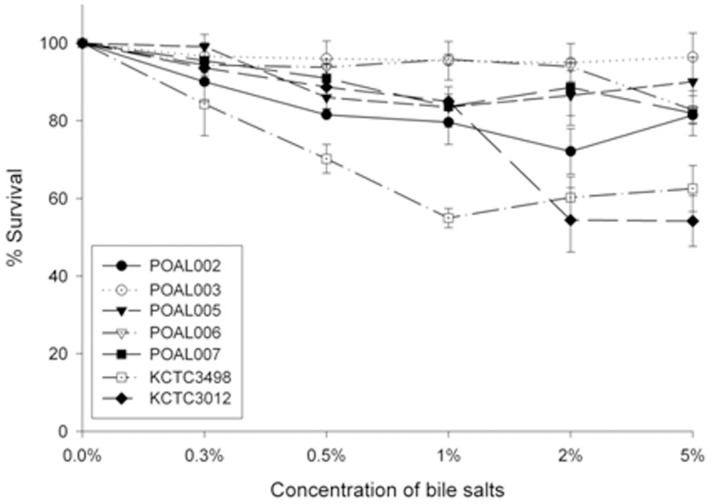
Effect of bile salt concentration on the viability of *L. brevis* POAL strains. Survival rates were estimated after 18-hours at 37°C in the indicated bile salt concentration.

The POAL strains exhibited greater tolerance to bile salt than the reference strains KCTC 3498^T^, and KCTC 3102^T^ (Figure 4). All the POAL strains maintained more than 80% of plain control solutions below 1.0% after 18 h at 37°C. The POAL003, 005 and 006 strains were especially resistant, with more than 90% survival at >2% bile salt. The reference strains were more sensitive to bile salt than POAL strains. Survival rates were estimated after 18 hours at 37°C in the indicated bile salt concentration.

#### Antibiotic resistance

The *L. brevis* POAL strains and reference strains KCTC 3498^T^, and 3012^T^ had specific resistance to various antibiotics (Table 3). All of the *L. brevis* strains were resistant to tetracycline, norfloxacin, sulfatrimethoxazole, colistin, and the common Gram-negative antibiotics. These strains were, on the contrary, susceptible to amoxicillin/clavulanic acid, chloramphenicol and ampicillin without discrepancy. However, each POAL strain also exhibited an individual antibiotic sensitivity profile. POAL002 strain was resistant to neomycin while others were not. POAL003 was moderately susceptible to oxytetracycline, while POAL006 was somewhat sensitive to streptomycin. In general, unique antibiotic resistance profile distinguished each of the *L. brevis* strains tested (Table 3).

**Table 3:** Antibiotic susceptibility profiles of the POAL strains*.

Antibiotics	POAL 002	POAL 003	POAL 005	POAL 006	POAL 007	KCTC 3498	KCTC 3102
Apramycin	I	+	+	S	S	S	+
Gentamicin	+	S	I	S	I	S	+
Cefotaxime	I	I	+	+	I	I	S
Tetracycline	+	+	+	+	+	+	+
Amox./Clav Acid†	S	S	S	S	S	S	S
Chloramphenicol	S	S	S	S	S	S	S
Neomycin	+	I	I	I	I	I	I
Norfloxacin	+	+	+	+	+	+	+
Colistin	+	+	+	+	+	+	+
Ampicillin	S	S	S	S	S	S	S
Sulfa-trimetho	+	+	+	+	+	+	+
Erythromycin	I	I	I	I	I	S	I
Oxyteracycline	+	I	+	+	+	+	+
Doxycycline	I	I	+	I	I	I	S
Ciprofloxacin	+	+	+	+	+	+	+
Streptomycin	+	+	+	I	+	+	+

*+ = resistant; I = intermediate; S = susceptible ^†^Amox/Clav acid; Amoxicillin/Clavulanic Acid.

### Probiotic Properties

#### Antibacterial activities

The five POAL strains were tested for their antimicrobial activities against various foodborne bacteria using the penicylinder method (Table 4). All the POAL strains demonstrated a broad spectrum of antimicrobial activity against some harmful enteropathogens. *C. jejuni and C. perfringens* were most strongly suppressed and *S. aureus, Salmonella* were second most. On thecontrary, the normal commensals such as *E. faecalis, B. cereus* were not affected at all. In case of *E. coli, a normal strain,* ATCC33694^ T^ avoid antimicrobial activities of the *L. brevis* strains while another non-pathogenic strain, ATCC 8739^ T^ was severely inhibited. Beneficially, however, the enteropathogenic O157:H7 (ACTC43888^T^) strain also was prevailed by the POALs. Similar to the reference strains, the antimicrobial properties of POAL strains did not coincide with Gram satiability.

**Table 4:** Antimicrobial spectrum of the selected *L. brevis* POAL strains*.

Indicator strains	*L. brevis* strains						
	POAL002	POAL003	POAL005	POAL006	POAL007	KCTC3498	KCTC3102
*Bacilluis cereus* KCTC 1012^T^	−	−	−	−	−	−	−
*Campylobacter jejuni* ATCC33560^ T^	++	++	+++	+++	++	++	++
*Clostridium perfringens* KCTC 3269^ T^	+++	+++	+++	+++	+++	+++	+++
*Escherichia coli* ATCC33694^ T^	−	−	−	−	−	−	−
*Escherichia coli* ATCC 8739^ T^	++	++	++	+++	+++	+++	+++
*Escherichia coli* O157:H7 ATCC43888^ T^	++	++	++	+++	+++	+++	+++
*Enterobacter aerogenes* KCTC 2190^ T^	+	+	+	++	++	+	+
*Enterococcus faecalis* KCTC 2011^ T^	−	−	−	−	−	−	−
*Klebsiella pneumoniae* KCTC 2208^T^	+	+	+	+	+	+	+
*Listeria monocytogenes* KCTC3567^T^	−	−	−	−	−	−	−
*Pediococcus acidilactici* KCTC 1626^T^	+	+	+	+	+	+	+
*Pseudomonas aeruginosa* KCTC 1750^T^	+	+	+	+	+	++	++
*Staphylococcus aureus* KCTC 1621^T^	++	++	++	++	++	++	++
*Salmonella typhi* KCTC 2424^T^	+	++	++	++	+	+	+
*Salmonella enteritidis* KCCM12400^T^	++	++	++	++	++	+	++

*Symbols for diameter of inhibition zones: +++ = >30 mm, ++ = >20∼25 mm, + = >15 mm; – = 0.

#### Intestinal survival of POAL strains

Another criterion for valid probiotic bacteria is viability in the intestines of live mammals. We investigated the viability of POAL strains in mice intestines, after 1 week of oral administration. After screening the bacterial colonies with pH 2.5 and tetracycline, we found that one strain, POAL006, survived in the intestine. The KCTC3498^T^ strain also passed the same selection, while another reference strain, KCTC3102^T^, failed. The identities of POAL006 and KCTC3498^T^ colonies were confirmed by RAPD analysis (Table 5).

**Table 5:** Viability of *L. brevis* strains in the mouse intestine after administration for 1 week.

Strains	POAL 002	POAL 003	POAL 005	POAL 006	POAL 007	KCTC3498	KCTC3102
Identified	−	−	−	+	−	+	−
Matched	0	0	0	100%	0	100%	0

#### Elevated expression of Glutamate decarboxylase (GAD)

**Figure 5:**
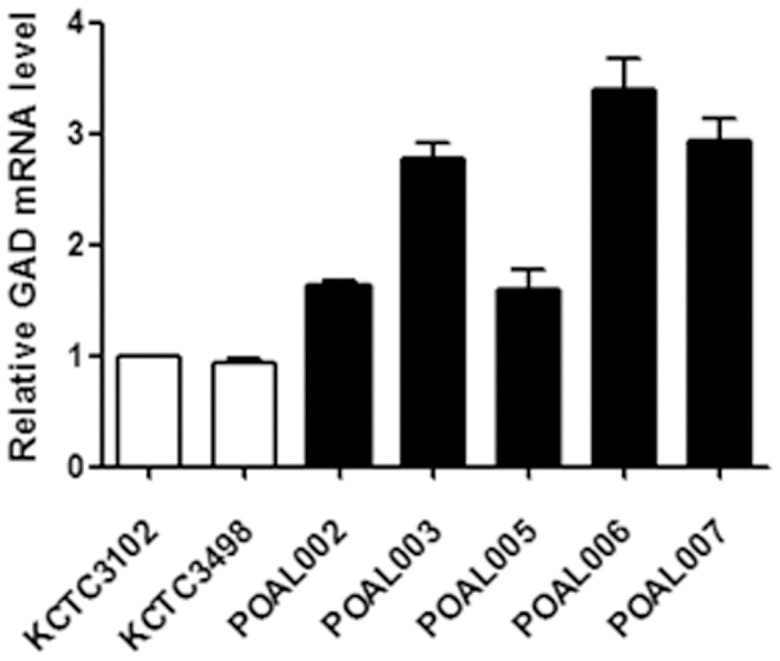
qRT- PCR analysis of GAD expression. Total RNA was isolated from *L. brevis,* converted to cDNA and analysed by qRT-PCR. KCTC3498^T^ and KCTC3102^T^ strains were used as references. Bars represent relative mean intensity. All values are expressed as the means ± SE, and the statistical significance was set at P<0.05.

All POAL strains expressed GAD mRNA at high level compared with the KCTC3498^T^ and KCTC3102^T^ reference strains. GAD mRNA levels in POAL006 were about three fold that in KCTC3102^T^. POAL003, 006 and 007 expressed two times more GAD mRNA than the reference strains (Figure 5). Higher expression of GAD mRNA suggested that the POAL strains are potential probiotics.

## Discussion

Despite its strong antibacterial properties, fermented of *Aloe vera L*. has been scarcely examined. It may due to the fact that effective ingredients in the leaf gel may be degraded after oxidation, and consequently, their antibiotic activity may be weakened to be hardly detectable. Because the fermentation reached at about pH 3.8, it is assumed that acid-tolerant bacteria will be found in the fermented stuff. Previous studies of the acidic fermentation of foods, plant-derived LAB such as *L. plantarum*, *L. acidophilus*, and *L. pentosus* were discovered, these observations led us to investigate LAB in fermented *Aloe vera*.

We identified five LAB strains in naturally fermented *Aloe vera* leaf flesh. API 50CHL analyses revealed that all LAB strains were *L. brevis*, and their identities were confirmed by RAPD pattern analysis and 16S rRNA sequence comparisons. These strains were unique in their acid and bile tolerance, resistance to antibiotics and GAD expression. We named these POAL (Probiotics Originating from Aloe Leaf) strains because they possess probiotic characteristics similar to those of the *L. brevis* reference strains, KCTC 3498^T^ (isolated from animal faeces) and KCTC 3102^T^ (isolated from pickles).

The five novel POAL strains identified in this study are all highly acid tolerant, surviving at pH 2.5 for as long as 4 hours and even longer(up to 6 hours) at pH 3.0. Generally, *L. brevis* strains do not survive at pH 2.5 [29], [37], and the reference strains used in this study, KCTC3498^T^ and KCTC3102^T^, did not persist for 2 hours at pH 2.5 (Figure 3). High acid tolerance may confer sustainability on these strains to survive in the gastric and intestinal environments. Another essential characteristic of probiotics is tolerance to bile salts, which are another barrier to LAB colonisation of the intestine; the probiotics should withstand the bile salts in the environment. All of the five POAL strains had above 80% survival rate after incubation for 18 hours in 5% bile salts. However, the reference strains’ survival rate was below 70%. Interestingly, strains POAL003 and 006 exhibited higher tolerance to both bile salts and strong acid, suggesting that these strains might survive in vivo passage to the intestine. In fact, viable POAL006 was recovered from the intestines of mice one week after ingestion (Table 5). Although no more strain detected in this assay, it may refer to the selection in, pH 2.5 for 4 hours, too stringent conditions. In the acid tolerance test, POAL003 survived at pH 3.0 but not pH 2.5.

Along with acid and bile tolerance, antibacterial activity is another valuable property of probiotics. The five POAL strains identified in this study inhibited the growth of many harmful enteropathogens while did not restrain most of normal commensals in the gut. Contradictory results against *E. coli* strains await further intensive investigation. Antimicrobial properties of POAL strains had no relevance to Gram stainability of enteropathogenic strains [22].

The existence of GAD may also validate the POAL strains. GAD is a pyridoxal 50-phosphate (PLP)-dependent enzyme, that catalyses the irreversible GABA [34], [35]. GABA is a well-characterised inhibitory neurotransmitter with hypotensive and analgesic properties. GABA also has tranquilising effect, particularly with regards to insomnia, depression and an autonomic disorder observed during the menopausal or presenium periods [16], [18], [23]. It induces insulin secretion from the pancreas and effectively relieves diabetic troubles [10]. Therefore, GABA attracts special interest as a potential bioactive component in foods and pharmaceuticals. In this study, GAD gene expression in the POAL strains was approximately two fold that in the reference strains (Figure 5). Several investigators have previously described GAD expression in LAB [27], [28]. Although direct analysis of GABA synthesis should be performed, these results suggest that our POAL strains may be resources for GABA production.

In conclusion, the five novel *L. brevis* POAL strains have many desirable probiotic characteristics such as antimicrobial properties against enteropathogens, a little inhibition to normal fauna, acid resistance, bile tolerance and intestinal viability. Their higher levels of GAD expression implied some potential validity. Antibiotic resistance also suggested their practical usage as supplements for patients with intestinal disorders or undergoing antibiotic treatment. Further studies of POAL strains will help us utilise these bacteria in pharmaceutical and industrial settings.
